# Ethical challenges and principles in integrated care

**DOI:** 10.1093/bmb/ldac030

**Published:** 2023-01-27

**Authors:** Alex McKeown

**Affiliations:** Department of Psychiatry, Wellcome Centre for Ethics and Humanities, University of Oxford, Warneford Hospital, Warneford Lane, Oxford, Oxfordshire, OX3 7JX, UK

**Keywords:** evidence, complexity, multidisciplinary teamwork, holism, specialization, ethics/professionalism, integrated care

## Abstract

**Introduction:**

Integrated care is an established approach to delivery in parts of the healthcare infrastructure, and an ideal which, it is claimed, should be realized system-wide. Its ethical weight derives from its defence of a view about how healthcare ought to operate. Although the goal of integration is laudable, it is ethically and practically complex, involving trade-offs.

**Sources of data:**

Considerable evidence attests to widespread enthusiasm for integration, given the need to prevent harm and extend the reach of scarce resources. Equally, evidence increasingly highlights the obstacles to successfully translating this ideal into practice.

**Areas of agreement:**

The principle that healthcare should be seamless, ensuring that patients do not come to harm through gaps in care enjoys broad agreement. There is a similar consensus that placing the patient’s perspective at the centre of decision-making is vital, since this enables identification of these gaps.

**Areas of controversy:**

Integrating care by making it seamless entails blurring boundaries of care domains. This risks undermining the locus of responsibility for care decisions via confusion about who has ownership of specialist knowledge where domains overlap. There is a lack of consensus about how successful integration should be measured.

**Growing points:**

More research into the relative cost-effectiveness of upstream public health investment in preventing chronic ill-health caused by modifiable lifestyle factors vs integrating care for people already ill; further research into ethical implications of integration in practice, which can be obscured by the simplicity of the fundamental normative principle guiding integration in theory.

## Introduction

Integrated care is both an established approach to organizing and managing *parts* of healthcare delivery and, commonly, an ideal which, it is claimed, healthcare professionals and institutions *should* aim to realize *system-wide*, as a guiding principle underpinning the whole of the healthcare infrastructure. To the extent that the exhortation for more integration is heard increasingly frequently, it is held by its proponents to have *ethical* weight, since it is presented as a view about how, in the interest of patients, healthcare *ought* to operate. Although the *goal* of integration is—to be clear—laudable and to be recommended, it is philosophically, ethically and practically complex, involving numerous trade-offs which, for reasons explained further on, required due consideration.

Straightforwardly, and using the definition offered by Singer et al. (p. 197), ‘*integration seeks to combine organizational parts into a unified, synergistic whole’.*^1^ So it is too in the specific context of healthcare.^2^^,^^3^ The vision communicated by the term ‘integrated care’ is one in which all parts of the healthcare infrastructure—different medical specialisms, nurses, allied health professionals; systems at the local, regional and national levels; primary, secondary, tertiary and social care—operate seamlessly, overcoming the naturally occurring delineations which distinguish one domain from another.

Integrated care, then, is an approach to healthcare that has emerged in response to potential or actual gaps in care where professional domains either do not overlap or do not have boundaries that cleanly meet their adjoining ones, over which continuity of care can be ensured. This may be due to: oversights in what is required to provide proper care; uncertainty about where responsibility for care lies in a patient with needs attended to by a range of specialists; or because of a lack of engagement with the patient about what they need; or any number of other reasons. In any case, the call to ‘break down silos’ will be familiar to anyone working in healthcare in the UK; and the driver of this call is to ensure that patient needs can continue to be met in those places where there is a risk that care could be disjointed.^4–6^

The call for integration has several roots and is characterized by interconnected economic and moral concerns. First, the healthcare burden is growing because lifespan has increased; and since people use disproportionately more healthcare the closer they get to the end of their lives and their needs become more numerous and complex, this growing burden must be offset, the sources of which include efficiency savings. Second, we are getting better at turning acute conditions into chronic ones which, again, prolongs life but increases economic pressure on scarce resources. Third, a function of advancing medical knowledge is increasing specialization, and this atomization of the body needs, somehow, to be recovered into an approach which treats the whole—that is to say, *integrated—*patient. Finally, each of these supports the overriding moral case: if integration would improve and extend care for more with the resources available, it *ought* to be done.

For the reasons outlined, therefore, integrated care is a normatively significant innovation in healthcare. While the goal of healthcare integration is one that should be pursed in light of the interconnected economic and moral case for it, the case in its favour nevertheless has *ethical implications* which it is important to unpack and absorb. These implications are dealt with in more detail over the coming pages, but we can summarize the challenge here by way of introduction.

Integration is one sense antithetical to the established direction of travel in medical understanding, and in this respect the two stand in tension. Tangible, measurable success in medical science is a relatively modern phenomenon and is, broadly speaking, both a product of the scientific revolution^6^ and consistent with the epistemic paradigm that characterizes it. This is to say, medicine’s route to reliable, actionable, *scientific* knowledge and, indeed, therapeutic success, has been to move away from the holistic practice of folk or indigenous healers which, though undoubtedly supportive of spiritual needs, was and is not clinically successful in the way that modern biomedicine has become.^7^ Instead, the decisive therapeutic advances have come through breaking the systems of the body into constituent parts—kidney, blood, lymphatic system, bones, brain and so on—and engaging in basic and applied science to understand how these systems operate. However, this method is in one respect *divisive,* since it *atomizes* the constituent systems of the person, and the body in which personhood is realized. Recognizing the trade-off that this entails, Shaw and Rosen (p. 61)^8^ characterize integration as a *wicked problem*:

*‘…Like other health care problems, the general increase in fragmentation reflects the increasing complexity of the health care system and increasing specialization. As diagnostic taxonomies and treatment opportunities have expanded, the challenge of coordinating care has increased’.*

A challenge posed by integration, therefore, is how to overcome this atomization and reunite these systems in a way that meets the unique care needs of the subjectively experiencing patient, while retaining the benefits that scientific atomization of bodily systems can bring. This is *ethically* significant because the tenets of specialization inform the training and career trajectory of healthcare professionals, which informs norms of decision-making authority in corresponding areas of practice.

To the extent that specialization can contribute to the problem that integration is deployed to resolve, integration can pose a challenge to entrenched assumptions about expertise and their boundaries, since one of its premises is that borders should be seamless, and thus *dissolved* to some degree. If the borders of domains of specialist knowledge are compromised, how are healthcare professionals to know what is definitively within their own domain rather than an adjoining one? Efforts at integration that are poorly planned, taught and delivered with respect to negotiating this question carry a risk of undermining professional identity and autonomy, the impact of which on professionals is significant.^9^ Crucially, a potential risk from ineffective integration is that patients may come to harm by falling through a different kind of ‘gap’; namely, one that follows from ambiguity over the locus of responsibility for care decisions.

## Sources of data

The call for better integration of healthcare is now resoundingly common. The most recent and prominent example of this has occurred in the context of the Covid-19 pandemic. The pandemic revealed numerous structural problems with the healthcare infrastructure, for example: transitions between primary and secondary care causing harm where the systems supporting transitions across this boundary were inadequate; vulnerable groups in care homes coming to harm due to inadequate processes governing discharge from hospital and other individuals coming to harm where measures to minimize the risk of transmission in different acute and long-term care settings were substandard. The pandemic brought into the foreground how some of its worst effects tracked pre-existing socio-economic, racial and cultural fault lines, where groups who were already vulnerable were at increased risk of harm because of gaps in care. It is deficiencies and inequities such as this that *successful* integration can help to reduce.

Irrespective of and prior to the pandemic, however, the call for integration and the evidence in support of it have been growing steadily for at least a decade. A prominent example of this was the UK Government’s Five Year Forward View for Health,^10^ which made the case for integrating health and social care. Subsequent to this, the UK’s Department of Health became the Department of Health and Social Care, with the functions of both previously distinct domains assumed under the remit of a single ministry of state. Similarly, a wealth of data has been produced to advocate for better integration of health and medical services across the National Health Service (NHS); and for a more holistic approach to health that takes into account social determinants, articulating an expanded view of what healthcare should encompass, alongside standard features such as diagnosis, referral to specialists, drug prescriptions and so on. Whether in the UK or elsewhere, a unifying theme in integration policies is the recognition of the need for holism if patients are to receive the right kind of support. As Glimmerveen et al. (p. 2)^11^ have noted:

*‘…the emergence of integrated care as a policy imperative signifies a growing concern with “the whole person” within the organization of care services. Following from the realization that people’s health and wellbeing are, for a large part, shaped by their broader social environment…improved connectivity between medical and social domains has become a defining objective within many pursuits of integrated care…The underlying implication is that care services need to be attuned to the ‘life-world’ of service users and citizens…and that professional and organizational efforts need to be aligned with people’s private responsibilities’.*

Alongside very visible headline instances of the case for integration, such as the Five Year Forward View for Health, numerous initiatives have been designed and implemented at local, regional and national levels,^12^ by a range of institutions, including: Clinical Commissioning Groups^13^; GP collaboratives^14^; individual NHS hospital Trusts^15^; local authorities.^16^ These have been bolstered by a stream of reports and studies from think tanks, charities, and health analysis institutions, including: The King’s Fund,^17^ the Health Foundation,^18^ the Nuffield Foundation,^19^ NESTA^20^; along with training schemes in integrated care for doctors and other healthcare professionals.^21^^,^^22^

Practical efforts such as these have also generated theoretical models of integration, which have in turn been adopted in subsequent integration projects. Examples include Suter et al.’s^2^ Ten Principles of Integrated Care, Valentijn et al.’s Rainbow Model of Integrated Care,^3^ Leijten et al.’s SELFIE Framework.^23^

All of this had led to the emergence of a self-sustaining ecosystem of inputs and outputs feeding integrated care theory and practice in both directions, and which continues to grow. Again, what unites these is a shared set of straightforward principles: that there is a pressing and combined moral and economic imperative to improve and extend patient care by reducing gaps between specialist domains and in doing so improving efficiency and saving money; and that this must, necessarily, be done by placing patient experience, views, wishes and autonomy at the centre.

## Areas of agreement

There are several areas of agreement about what integration is and why it should be pursued as a guiding principle for healthcare management, and we cover some centrally relevant of these here, namely: the general principles underpinning integration; the *prima facie* case for integration; a conceptualization of integration as a necessary response to the increasing fragmentation of healthcare along lines of specialisms; the inevitability that healthcare will increasingly need to be conducted in a way that presumes multidisciplinary teamwork to ensure that gaps in care are eliminated; and, consistent with the move away from paternalism to a shared decision-making model, that implicit to the philosophy of integration is a view in which the patient’s experience should be at the centre of those options and thus explicitly sought. Hughes et al. (p. 449)^24^ offer the following clear articulation of this position:

*‘The patient perspective is generally considered to be the organizing principle of integrated care…From the patient perspective, organizational divides between health and social care can cause duplication (e.g. multiple assessments by different services) or gaps in care when transitioning from one setting to another (e.g. from a hospital to a home or community). Specialization of health care treats each medical condition separately, with increased potential for fragmented, suboptimal care that fails to consider interrelated health and social care needs. Person-centered and coordinated care is intended to address such fragmentation and to “activate” patients to engage in care planning, decision making, and self-management’.*

To begin, there is broad agreement about what the principles underpinning the project of healthcare integration are and should be. We know from qualitative academic research and evidence from Patient and Public Involvement (PPI) programmes that patients, fundamentally, do not wish to experience gaps in care. Therefore, given the primacy of patients’ needs, if integration helps to reduce gaps, then it is an approach which is consistent with this foundational ethical commitment of care. There is also a consensus that an era-defining challenge of contemporary healthcare is the dual challenge of increasing successes in prolonging life and treating disease. Since this creates economic pressure on scarce resources, if we wish to protect the gains to health and longevity, we will, collectively, have to find a way to accept the trade-off; namely, finding ways to make scarce resources go further. Indeed, a striking feature of the discourse about healthcare integration is how little dissent there is about whether, *as a matter of principle,* it is an approach that should guide the organization and delivery of healthcare. As Garattini et al.^25^ note:

*‘IC has become a sort of philosophical concept over time, on which it is really hard to disagree. Integrating services when people need care should be a common feature of high-quality health systems’.*

Part of the reason for this is that once a patient moves beyond the straightforward resolution of a health problem by their General Practitioner (GP), the patient necessarily becomes part of a *network* of health professionals, given that all specialisms have limitations to what problems they are equipped to address. Indeed, even GPs, whose *specialism* is, by definition, *generalism*, will be deficient with respect to more detailed knowledge about particular systems of the body, hence the referral system and the division between primary and secondary care. A naturally occurring ceiling on what any one professional can learn and specialize in is an immovable constraint and, as such, there can be no dispute that expertise in the whole person must be distributed across numerous specialists. With this in mind, Plochg et al. (p. 21)^26^ have captured the sense of inevitability that characterizes the move towards healthcare integration in recent times:

*‘Multi-morbidity cannot be managed by isolating and treating each morbidity by linearly organized specialist interventions anymore, due to complexity, interrelation with other diseases and strong correlation to socio- economic conditions’.*

Here again, then, a logical feature of contemporary care is evident; namely, that care for the whole patient *must and can only* be a *shared* endeavour. This necessarily requires a presumption of effective multidisciplinary teamwork, with the patient’s experience at the centre as a guide for how the right clinical response is agreed jointly and in collaboration.^27^ Taken together, these considerations give some idea of the theoretical robustness of the case for integrated care and indicate why it secures such widespread agreement.

It is worth expanding on the last point here. The centrality of the patient’s experience leads us to those aspects of healthcare integration which are more problematic or controversial. A major change in the orientation of healthcare in the post-war era has been a move away from medical paternalism, towards a model which emphasizes the central ethical significance importance of patient autonomy, with these two sets of expertise balanced in a model of *shared* decision-making. While it would be irresponsible of a doctor to meet literally any and all wishes of a patient at any health cost to the patient, given that *consent* is vital for the moral legitimacy of medical intervention, dialogue with patients towards understanding the options available to them and agreement on one chosen *by the patient* is in the foreground of contemporary norms of practice. This, again, is logically consistent with the case for integration. The uniqueness of individual subjective experience means that only by soliciting that experience can it be known what the gaps and deficiencies would be for a given patient. As such, the solicitation of those experiences is an indispensable, necessary constituent of effective healthcare integration.^28^

These justifications sound rational and straightforward. However, realizing successful integration in practice is more complicated than the clarity of its principles suggest. As Goddard and Mason (p. 2)^29^ note, because of the degree of agreement about the theoretical basis for integration, ‘*it is…easy to be convinced of the approach without questioning the basis of the evidence’.* Indeed, a body of research has emerged in recent years which has started to produce evidence that articulates why practice is more complex and controversial than the broad agreement, which is enjoyed by integration theory.^30–33^ In the next section, we therefore consider some of the practical obstacles and controversies that occur when integration is put into practice.

## Areas of controversy

Despite considerable agreement about the value and importance of healthcare integration, it has implications that are potentially ethically problematic and/or controversial. These might be broadly summarized as: consequences of integration for professional autonomy and responsibility^33^; a lack of consensus about how to *measure* the effectiveness of integration,^34^ not least because measurement itself may be a complex task in its own right^35^; and practical and financial obstacles to institutionalizing integration. Given the widespread enthusiasm for integration, there is a case to be made that the first two of these have received insufficient attention in spite of the controversy that they reveal. This is a risk for the adequacy of integrated care systems, not least because of a lack of agreement about what outcome measures indicate the success of such systems. Indeed, a pertinent question to ask about integration is what can account for the difficulty of achieving something based on principles that are uncomplicated and widely agreed upon. As Lewis et al. (p. 6)^36^ note:

*‘The last 12 years have seen a determined but restless effort to test out ways in which integrated care might best be designed and implemented…Given this level of activity, one might question why the debate about how best to integrate health and social care in England remains unfinished business. No single programme has been able to distil key, generalisable “lessons” that have then been applied subsequently’.*

In the same way that it is axiomatic to integration that contemporary care can only be a shared endeavour, so there are similarly ineluctable consequences for professional autonomy in the context of integrated care.

Specialisms are characterized by particular fields of localized, detailed, knowledge about a particular bodily system. The more one has trained in a particular specialism, the more jurisdictional autonomy one will have over that domain, with respect to the particular needs of a given patient. However, since the person is a whole and their parts are interconnected, we cannot, logically, assume that the knowledge pertaining to each particular system is clearly delineated (indeed, it is partly in view of this that integration is needed). As Brown et al.^37^ (p. 433) articulate, attention to this theoretical aspect gives a vital insight into the nature of the challenges posed by care integration, since ‘*theorising about boundaries…helps to explain the concerns about role blurring and the lack of firm boundaries of responsibility’.*

It is at the borders of domains of knowledge and expertise, where specialisms meet and overlap, that knowledge is most contestable. As such, it is the borders between specialisms or domains of knowledge that risk causing uncertainty about which specialism has authority over a particular decision that has to be made. This is important, given the expectations that healthcare professionals hold about the remit of their role and ownership of a body of knowledge to which they have committed a great deal of time, effort and which is, in many cases, closely tied up with their personal identity.^38^ In spite of the fundamental strength of the ethical case for integrated care, when giving proper consideration to what it demands, it is equally fundamental that an answer is needed to *how* jurisdictional autonomy is to be negotiated and agreed in a system which has as one of its key presumptions that there are no clear borders between domains of care. McNeil et al. (p. 301)^39^ summarize this risk, noting that *‘…despite the inherent logic in interprofessional working, the reality is fraught with obstacles…these barriers relate to the overarching theme of professional identity’.* The more that integration is emphasized and characterizes the direction of travel of the healthcare infrastructure—indeed, given that the point of integration is to erode and overcome hard boundaries—the more persistent and relevant this challenge will be. As McKeown et al. (p. 9)^40^ point out, since the maintenance of hard boundaries is the antithesis of integration, so it follows that the prospect of successful integration is undermined by the highly autonomous practice that characterizes those boundaries.

Of course, we might be sceptical here as to how important this is. Even in an integrated system, doctors and other professionals will still have ownership of a significant body of specialist knowledge even if they are expected to relinquish, or share, some of this towards the edges. However, it is important in view of the primary objective of care, since, whatever else healthcare professionals prioritize—including the boundaries of their role with respect to the deployment of their own hard-earned specialist knowledge—what must always take priority is the patient. As Wackerhausen (p. 457)^41^ has summarized:

*‘The “caring and healing professions” – like nursing, medicine, physiotherapy, psychology, social work, dietetics, etc. – differ in numerous ways, but are nonetheless united by a shared goal: to do what is best for the patient. This is their raison d’eˆtre; it is their official reason for existence’.*

A key feature of successful prioritization of the patient in a multi-specialist care environment is, as we have seen, being able to ensure that none of their needs fall into a gap between two or more of those domains (and, indeed, we also know now that it is *because* of the risks posed by these gaps that integration is so strongly advocated for). However, the corollary of uncertainty about how to agree the boundaries of professional *autonomy* is commensurate uncertainty about how to agree where *responsibility* for care judgement and decisions lies. The right to a body of specialist knowledge is grounded in the assumption that one is able to take responsibility for decisions made based on that knowledge. But this relationship is, at least potentially, compromised if there is any doubt about in whose domain authority for a decision lies, and indeed this poses a potential threat to the success of integration, as Jolanki et al. (pp. 252–253)^42^ have identified:

*‘…unclear definition of roles and responsibilities of different actors within primary care and specialised care as well as between health and social care stall integration’.*

This is a potential challenge for successful healthcare integration because uncertainty about who should take responsibility for a particular decision, risks undermining what is meant to be a key advantage of integration; namely, that gaps in care are closed. Where no protocol exists for agreeing on responsibility where domains overlap or meet, where decisions must be made, a patient may come to harm. If this occurs, the system will collectively have failed to effectively prioritize meeting the needs of the patient, but it may be unclear within this system who should be held responsible. This in turn undermines the corresponding right of specialists to the nominal authority over the particular domains of knowledge that they are assumed to have.

In an environment which is characterized by under-resourcing, relative to a growing healthcare burden, there are grounds to be concerned about this. Two important and interconnected reasons are clinical negligence and risk aversion. The fear of the former leads to the latter and makes ‘passing the buck’ for medical decisions more likely.^43^ Crucially, although integration may be invoked as necessarily a solution for escaping the feedback loop between the two,^44^ for reasons we explain, this is potentially misleading.

NHS Resolution statistics^45^ reveal that clinical negligence claims against the NHS have risen substantially over the past fifteen years—from 5426 in 2006/7 to 12 629 in 2020/21. Similarly, Lane et al.^46^ report National Audit Office statistics which confirm that clinical litigation payments quadrupled over a decade between 2006 and 2016, from £0.4bn to £1.6bn. Given this dramatic escalation, it is unsurprising that a growing culture of risk aversion in the NHS has been identified. A consequence of this, as Anandaciva et al.^47^ identify, is a corresponding increase in health professionals ‘passing the buck’ in responsibility for clinical and care decisions.

In response to the risks associated with failures to take responsibility, a refrain from numerous NHS Trusts has been for more integration^48^^,^^49^ to tackle the failures caused by passing the buck reported by patients.^50^^,^^51^ This makes theoretical sense: the possibility of passing the buck implies that whoever is doing the passing can appeal to a boundary between their domain of expertise and another and the corresponding jurisdictional dividing line that this represents, in virtue of which they can argue that they do not bear responsibility for whatever was or should have been done. However, the argument in the opposite direction makes equal theoretical sense.

It can be argued that it is just as easy to pass the buck in a system that is apparently integrated as it is in one that is not. Imagine a dispute about responsibility between clinician A who is a specialist in domain X, and clinician B who is a specialist in domain Y. The dispute is about harm resulting from poor decisions that failed to ensure continuity of care from X to Y, but it is also *acknowledged* that in certain respects, and for reasons already outlined, domain X *is integrated with* domain Y. According to the principles of integration, the dissolving of the nominal boundary between domains X and Y *should* help to prevent harm, because it follows from these principles that clinicians A and B *will necessarily share* responsibility for decisions occurring across that previously recognized boundary. As Kumpunen et al.^52^ note, though, we cannot necessarily help ourselves to this assumption.

In this case, for example, clinician A can *still* claim that responsibility should lie with clinician B. As much as a specialist might be unwilling to relinquish jurisdictional autonomy in their domain, it is equally plausible, if they feel vulnerable due to a negligence claim brough by a patient, which they might be inclined to pass the buck to the specialist operating in the contested or shared region in which the decision was made. There is nothing about the integration of the domains *per se* which guarantees that disputes will not occur.^53^ Crucially, here, whether the integrated region in question is understood as ‘contested’ or ‘shared’ depends on one’s point of view and is likely to be at least partly informed by what one has at stake in terms of the possible consequences, should any legal action be successful. As such, unless it is established concretely *how* integration is to be done, there is a risk that it will not *in practice* adequately solve the problem that it is meant to and which it can solve in theory.^54^

It is important to be clear here that the scenario outlined here does *not* show that integration should *not* be pursued or that it is somehow incoherent. Rather, its purpose is to highlight the potential discrepancy between apparently clear, simple, straightforward principles and the complications that may follow from their application, because the *mechanisms* by which their application is attempted are numerous and diverse and may be under-specified.^24^ The guidance referred to above from NHS Trusts, which stipulates that staff should not pass the buck is no doubt necessarily and laudable, but, as with guidance elsewhere, it does not properly articulate *how* this is to be done.

Despite the wealth of guidance, integration is clearly hard to achieve. It is easy to sign up in principle to admirable statements of standards since these are clearly hard to disagree with; but they do not guarantee success in practice. As with the associated concept of ‘person-centred care’, integration is *aspirationally* important for the NHS^55^ (and, presumably, healthcare systems in other countries which face similar challenges to the UK). But there is more to be done to help realize the indisputable vision. In summary, the mechanisms according to which responsibility *is in fact* shared still need significant development so that this can be achieved successfully.^56^

The final area of controversy which should be highlighted follows from the challenge outlined above. This is the scale of the logistical challenge that must be met in realizing the vision of a fully integrated healthcare system. Healthcare integration is rhetorically appealing and, indeed, even under scrutiny it is difficult to cogently argue that it is not, ethically speaking, the goal that should be pursued if we wish to properly meet the needs of patients. However, it is arguable that the difficulty in rationally refuting the case for integration obscures the practical obstacles to realizing it.^52^ There are several reasons for this.

First, the move to a presumption of shared and overlapping domains of specialist knowledge will require a profound reorientation of medical training and the professional assumptions that guide it. Since health professionals may work for up to four or five decades, this means that the transformation required will be generational in terms of its timescale. Second, and relatedly, however much consensus there might be about the need to ‘break down silos’ and ensure that transitions in care are seamless, the transitions represent nominally bounded and discrete domains of knowledge, and these domains will continue to exist even in a well-integrated system. Third, the heterogeneity of healthcare professions, the commensurate diversity of assessments of cost-effectiveness, and the uniqueness of each individual’s situation—in virtue of which the patient’s experience must be central to integration—means that there is no uniform way to measure success.^57^ As Berntsen et al. (p. 11)^58^ summarize:

*‘The translation of the overarching goal into relevant and realistic goals of care is a complex negotiation and balancing act.’*

Fourth, and finally, more evidence is required for how integration is to be done at scale. This is to say, while numerous relatively localized integration schemes have demonstrated success, the complete integration of all services pertaining to healthcare at a national level remains a formidable challenge. Although achievable over time, this is, again, a project which will occur over decades and which will, unavoidably, require the relevant institutions to invest in the production of the evidence for how it is to be done, as Raus et al. (p. 6)^59^ point out:

*‘…in the short term, promoting collaboration and networks in fact costs money before there is any pay-off…Policy-makers who wish to implement policy need to commit to providing sufficient resources to make that policy work’.*

Nevertheless, evidence is being produced steadily and as this accumulates it will continue to increase the body of knowledge about the precise mechanisms by which integration can be successfully achieved. In turn, as this continues, the scale at which it can be achieved will continue to increase. However, a combination of the need to overturn previous operational norms and the assumptions of the numerous individuals working within the healthcare infrastructure places a constraint on how rapidly coherent system-wide integration can be realized. As Stoop et al. (p. 1136)^60^ have observed with respect to the evidence-related challenge:

*‘…empirical evidence for the effectiveness and cost-effectiveness of integrated care is still inconclusive, partly due to the heterogeneous nature of the integrated care sites and/or the use of different outcome measures’.*

For this reason, it is important to keep in mind that the sheer complexity of the enterprise behind the cogent and elegant theoretical underpinning of integration will be a limiting factor on the speed at which the infrastructure can be transformed. Crucially, we can return here to the point raised earlier about the historical specialization of modern medicine representing a move away from indigenous or folk healing practice which enables and tracks inexorably the advances in knowledge made by discrete fields of scientific enquiry. To the extent that it attempts to reconcile this atomization with a recovery of the whole patient, integration can be seen as a way to revive the key ethical feature of these more scientifically primitive practises. However, this is a task the conceptual and practical difficulty of which should be recognized. As Reach (p. 455)^61^ has observed:

*‘Even if it is a return to the ancient, this move may represent a change in paradigm that will be, as always, slow and progressive, and whose acceptance will certainly meet resistance’.*

Taking all of the above into account, therefore, it is clear that there is work to do both in areas where there is principled agreement about the value of integrated care, and in areas where there is a spread of opinion about how it should be delivered or how its trade-offs should be negotiated in practice. The final section, therefore, outlines some potential future avenues for further research.

## Areas timely for developing research

Healthcare is one of many areas of life in which there is much to learn from the Covid-19 pandemic. Some of what can be learnt is directly relevant to integrated care, but crucially, *not only* in relation to Covid-19 *specifically*. Rather, the pandemic has revealed pre-existing weaknesses across the healthcare infrastructure, which it is a pressing ethical priority to be addressed, even once the pandemic recedes and the unique pressures that it has exerted abate. As Stein et al. (p. 2)^62^ write:

*‘Covid-19 has cruelly exposed the fact that those people who would most benefit from a coordinated response to their needs are the least likely to receive it. This is now likely to get worse, not better, without concerted action. Covid-19 should thus be seen as a wake-up call to radically change the policies on health and wellbeing’.*

A salient feature of the pandemic has been its highlighting of how deeply structural social, economic, cultural, and racial inequities are embedded in the UK and elsewhere. Covid-19 has caused disproportionate harm to people already experiencing various kinds of disadvantage—whether this is because they are women, or they are precariously employed, or because they are members of an ethnic group with historical reasons to be suspicious of medical authority, or combinations thereof, among numerous other reasons—and the state has a responsibility to act in such a way that brings proportion in the event of another pandemic. This presents an opportunity for integrated care research in (at least) two ways.

First, we know that one of the economic drivers of the call for integration is a rise in chronic conditions, some of which could be mitigated by better, more sustainable lifestyle choices; and the empowerment for people to do this will need to come to some extent from government investment in the opportunities and education that gives people the knowledge about how to do so. There is, therefore, an important programme of health economics research to be done in establishing the relative cost and benefit of different allocation models for health funding, as Rocks et al. (p. 1218)^63^ have identified:

*‘…directed expansion of health economics towards the evaluation of integrated care is necessary to ensure decisions surrounding the implementation of integrated healthcare delivery are likely to benefit, rather than hinder, aims to meet increasing demands on tightening budgets’.*

Given finite resources, it will be important to establish what proportion should go towards developing the evidence for healthcare integration and the means to achieve it, set against what is required to reduce the structural inequalities that conduce to poor health and the increased strain on the healthcare system caused by modifiable social determinants of health. Clearly, since resources and time are finite, not everything desirable will be achievable, and a priority-setting exercise will therefore need to be done which takes into account both the value and need for healthcare integration in the interest of providing seamless care, and how much strain could be taken off the healthcare infrastructure if the government increased investment in public health.

Second, and intersecting with this programme of research in health economics, greater and more detailed engagement with the ethical and philosophical dimensions of integration, in the context of the tensions and inequities incidentally revealed by the pandemic, is urgently needed. Despite the inherently and acutely normative nature of healthcare integration, surprisingly little work has been done *in ethics* about its implications and to unpack and scrutinize its guiding principles. It is easy, given the strength of the case for integration, to assume that it contains no ethical tensions or complexities, or that all of assumptions are explicitly understood and agreed upon. For the reasons outlined here, however, it is not obvious that this is the case. How, fundamentally, is the impossibility of one health professional being able to fulfil all healthcare needs, to be overcome, beyond the rhetoric that healthcare professionals should work together? What does the call for integration tell us about the nature of medical specialisms and how can we know what delineates one from another?

## Concluding remarks

Undoubtedly, given the complexity of a project that aims at full integration across the whole healthcare infrastructure, there are numerous potentially fruitful avenues for research, some of which will follow from the issues outlined in the review. As such, it is not possible here to have given an exhaustive list of suggestions in the space available. Nevertheless, the suggestions in combination with what has been outlined in the review should yield a clear overall conclusion. This is that healthcare integration is an idea whose time has come and that should be pursued, for interconnected ethical and economic reasons, given contemporary challenges, to prevent harm to patients. However, the practical delivery of a straightforward organizational principle is formidably complex, for reasons relating to the negotiation of specialist boundaries and the historical norms of healthcare practice that are embedded in the training of professionals.

With this in mind, the two matters suggested above function both as suggestions for what it would be timely to address, given the contingencies of the present historical moment, and as concluding remarks about the challenges that beset integrated care, based on the analysis presented. Pursuing the avenues of enquiry raised in the review, and others that emerge from close scrutiny along the lines set out here, will help to produce the evidence needed for improving the translation of elegant integrated care theory into effective integrated care in practice.

## Data Availability

No new data were generated or analysed in support of this review.
